# Pandemic disruptions: The subversion of neoliberalism

**DOI:** 10.1177/1473325020973344

**Published:** 2021-03

**Authors:** Nancy Ross

**Affiliations:** School of Social Work, Dalhousie University, Halifax, Nova Scotia, Canada

**Keywords:** Violence, women, children, violence against women and children, antiblack racism, neoliberalism

## Abstract

The implications of the COVID 19 pandemic signal both tragedy and possibility. This reflective paper considers the amplification of the concurrent pandemics of violence against women and children and anti-black racism during the responses to COVID 19 and renewed calls to action. The enforced ‘pause’ as a result of social isolation or distancing measures in response to COVID-19, has led many people to re-imagine a different world and ignited social movements across the globe. Education must inspire a vision of what our world could be and define what action is needed and the steps required to implement change. The critical reflection that characterizes most social work educational programs can provide opportunities to harness such imaginings in redefining ‘the possible’ in the quest for a more equitable and safer world. This article describes the potential of the pandemic to subvert the pervasive influence of neoliberalism by promoting collective notions of care.

The implications of the COVID 19 pandemic signal both tragedy and possibility. This reflective paper considers the amplification of the concurrent pandemics of violence against women and children and anti-black racism and renewed calls to action. The enforced ‘pause’ as a result of social isolation or distancing measures in response to COVID-19, has led many people to re-imagine a different world and ignited social movements across the globe. The critical reflection that characterizes most social work educational programs can provide opportunities to harness such imaginings in redefining ‘the possible’ in the quest for a more equitable and safer world. At no other time in recent history has the call for social justice been so insistent.

During the first few months of the pandemic the media posted news stories of the largest mass shooting in Canadian history, depicted images of global marches against anti-black racism and described marked increases in violence against women and children all while social isolation or distancing measures were enforced. These early months of the pandemic were characterized by hoarding and fear and at the same time acts of generosity and a swiftness of innovative responses unimagined before the pandemic. Examples included new housing options for the homeless, releasing inmates from prisons and a renewed call for equitable access to a living wage for all. This article describes these innovative responses as disruptions and discusses the potential of the pandemic to subvert the pervasive influence of neoliberalism by promoting collective notions of care.

The guiding political philosophy in Canada since the 1980’s has been neoliberalism (Brodie, 1996). It has been described as a regime change that facilitated a transition to corporate rule in which it is assumed that increased productivity and economic growth will improve human well-being (Donnan, 2014). Relying on a logic of a free-market economy and reflecting a small government approach, neoliberalism has resulted in a reduction of the welfare state and its responsibilities. Students at schools of social work across North America and beyond are taught about the implications of neoliberalism and the ways in which this mentality of government trickles down to order and influence our lives. Since the 1990’s there has been an intensification of neoliberalism that confers advantage to those already privileged by power and social standing and further disadvantages those who experience gendered, racial and colonial inequalities (Donnan, 2014). Individuals who are successful in the labour market attribute their success to their personal efforts and consequentially those who are unsuccessful feel inadequate. A sense of inadequacy and not quite measuring up is pervasive among university students who are steeped in a competitive environment that promotes individualism. Paradoxically, schools of social work extol relational connection and empathic communication, furthering a dissonance experienced by many students within educational contexts.

It is difficult to quantify the pervasive influence of neoliberalism and the myriad ways in which it gets ‘under our skin’ to order our thoughts about what constitutes our success and in turn influence our behavior; monitoring our appearance, employment and accumulation of possessions. As one student shared in class this past year, she felt pressure to be perfect and believed this pressure contributed to high rates of anxiety among her peers. But how is perfection defined? If we turn to Foucault’s concept of panopticism, in which he described the ways inmates in prison are constantly visible, subjected to surveillance and controlled seemingly by invisible authority figures, it can serve as an analogy regarding the impacts of neoliberalism. Facebook, twitter and other forms of social media comprise mechanisms of visibility evident in the consumer culture spaces, images and screen culture (Place and Vardeman-Winter, 2013). Invisibly the long reach of neoliberalism influences a new productive form of governance generated by these invisible forces running through the living mass of population. Amplified during the pandemic, consumption increasingly takes place in a digitalised milieu such as the internet, in which purchases and other interactions are monitored by businesses, for example, yet remain invisible to the vast majority of consumers. At the same time the massive explosion of social media sites working off the fear of invisibility ensure visibility (Place and Vardeman-Winter, 2013). Foucault’s description of the panopticon serves as metaphor to elucidate the ways in which neoliberalism results in both external and internal surveillance. As noted by Brown (2016) neoliberalism objectifies people as it reforms “subjective identities into narrow market actors in a world of human capital” (p. 3). In day to day life we become less connected to history, place, tradition and community, making this day and age feel particularly groundless.

Breitkreuz (2005) describes the pervasive influence of neoliberalism as a moving away from a model of social citizenship that recognized all citizens were universally entitled to benefits to a model of market citizenship in which those individuals unable to perform in the labour market are devalued and marginalized. I suggest the reverberations of the ‘pause’ caused by social isolation in response to COVID 19 is the potential to reverse this trend by reinstituting a model of engaged social citizenship that reimagines and transforms life in Canada. Nowhere is the call to transformation more insistent than in our responses to the pandemics of violence against women and children and anti-black racism which I discuss below to highlight the need for unflinching reflection accompanied by action.

Globally, at least one third of women experience physical or sexual violence (World Health Organization, 2013). Over half of the children in the world, estimated at 1 billion, experience the most severe forms of violence, between the ages of two and seventeen, each year (Hillis et al., 2016). Yet both forms of violence are shrouded by silence, secrecy and denial. However, for those who work in the anti-violence field the awareness of the pervasive nature of interpersonal violence is a daily burden. This knowledge contributed to the words of the director of the Transition House Association of Nova Scotia who noted she was in no way shocked to hear mainstream media confirm that violence against women lay at the heart of Canada’s largest mass murder in April of 2020 (Nourpanah, 2020). These tragic murders devastated Nova Scotians and unsettled the depictions of this province as a peaceful, welcoming and safe place to live. That underneath this veneer laid the possibility of such horror was unthinkable to many. However, as difficult as this mass shooting was to comprehend Nova Scotians and the entire country are attempting to understand what led to this event and what might have prevented it. In the continued analysis of this tragedy the issue of gender-based violence remains at the forefront. Those who work in the field of anti-gender-based violence hope the aftermath of this crisis will result in increased awareness of the scope of this problem and increased funding to address the urgent need for societal change. A peacebuilding analysis applied to violence against women and children recognizes structural and cultural ‘violences’ are inextricably linked to direct experiences of violence (Galtung, 1990, 1995). This implies that our cultural values and structural inequities serve to sustain current rates of violence against women and children. An international consortium of researchers identified reducing poverty and obtaining wage equity for women as a first step in responding to violence against women and children during a pandemic (Peterman, 2020). In order to do so the individual, highly competitive and alienating values of neoliberalism must be subverted and replaced by a society that prioritizes collective care and concern for those experiencing inequity and oppression. The emblem of Nova Scotia stating ‘Strong Together’ implies that our response to violence must be collective and implicates societal and political responses. The Executive Director of the Be the Peace Institute, Sue Bookchin (2020) recommends these responses be infused by an intersectional and feminist analysis. She recommends the provision of wage parity in every industry; the mandatory presence of women on corporate boards; more equitable access to capital for women-owned businesses; and school curriculums that teach children about healthy gender roles and relationships at every age as necessary responses. This small non-profit organization in Nova Scotia serves as an example of the ways in which neoliberalism can be subverted by a grassroot organization that works to promote community and coordinated system responses to ending men’s violence against women in service of a more just and peaceful world for all.

The call to end anti-black racism also includes the subversion of neoliberal ideologies and practices to redress years of neglect and indifference by providing support and resources to African Canadian communities. Canadian Senator Wanda Thomas Bernard (2020) has called for collective action to respond to the pandemic of racism. She indicates this will entail removing denial about Canada’s history of racism that results in the underestimation and undervaluing, and over-criminalization of African Canadians. She describes the cumulative impact of racism-related stress: “As an African Canadian mother, wife and grandmother of two young black boys I bear the burden of stress and worry of ‘living while Black’” (Bernard, 2020). Bernard is a former professor of social work who has written about how cumulative experiences of racism have serious health consequences for African Canadian communities (James et al., 2010).

Saad (2020) also acknowledges the need to remove denial about the history of racism and states it is time for white folks to engage in some radical truth-telling about our complicity in white supremacy and recommends that this engagement start as personal work. She invites a critical reflection that scrutinizes white supremacy as a system we are born into that informs the foundation of societal norms, rules and laws and grants unearned privileges, protection and power and is designed to keep you asleep and unaware of the repercussions for those who do not hold white privilege.

The murder of African American George Floyd by police officer Derek Chauvin has awoken collective rage that demands action. As the fall university term begins it is more imperative than ever that the individualism that marks neoliberalism be replaced by collective concern and action. Education should inspire a vision of what our world could be and define what action is needed and the steps required to implement change. The pairing of reflection followed by implementation of action is required. This moment of subversion of neoliberalism compels a re-imagining of what our social and community life could be if a more just and peaceful world was the measure of success.
